# Patient and Public Involvement in Paediatric Pragmatic Randomized Controlled Trials: A Mixed Methods Study

**DOI:** 10.3390/children12121638

**Published:** 2025-12-02

**Authors:** Shelley Vanderhout, Shipra Taneja, Pascale Nevins, Stuart G. Nicholls, Beth K. Potter, Maureen Smith, Alicia Hilderley, Dean A. Fergusson, Colin Macarthur, Monica Taljaard

**Affiliations:** 1 Institute for Better Health, Trillium Health Partners, Mississauga, ON L5B 1B8, Canada; 2 Institute for Health Policy, Management, and Evaluation, University of Toronto, Toronto, ON M5S 3M2, Canada; 3 Clinical Epidemiology Program, Ottawa Hospital Research Institute, Ottawa, ON K1H 8L6, Canada; 4 Methodological and Implementation Research (MIR) Program, Ottawa Hospital Research Institute, Ottawa, ON K1H 8L6, Canada; 5 Office for Patient Engagement in Research Activities (OPERA), Ottawa Hospital Research Institute, Ottawa, ON K1H 8L6, Canada; 6 School of Epidemiology and Public Health, University of Ottawa, Ottawa, ON K1N 6N5, Canada; 7 Patient and Public Involvement Partner, Canada; 8 Faculty of Medicine, University of Ottawa, Roger Guindon Hall, Ottawa, ON K1H 8M5, Canada; 9 Child Health Evaluative Sciences, SickKids Research Institute, Toronto, ON M5G 1E8, Canada; 10 Department of Pediatrics, Temerty Faculty of Medicine, University of Toronto, Toronto, ON M5S 3M2, Canada

**Keywords:** patient and public involvement, pragmatic trials, patient engagement

## Abstract

**Highlights:**

**What are the main findings?**

- In a survey of paediatric pragmatic trialists, just over half reported that they had con-ducted patient and public involvement (PPI) in their research.
- Benefits of PPI included better intervention feasibility, research quality, recruitment and retention, and applicability of findings, while barriers to PPI included insufficient resources, time, and training for building partnerships, especially with children, youth, and caregivers.

**What is the implication of the main finding?**

- Institutions and funding bodies need to invest resources and infrastructure in PPI partnership, including guidance to support researchers as they navigate identifying, preparing, and providing opportunities for children, youth, and parents as PPI partners.

**Abstract:**

Background/Objectives: Patient and public involvement (PPI) in the design, conduct, and dissemination of pragmatic trials may make trial results more relevant and meaningful. The nature of PPI in paediatric pragmatic trials has been poorly characterized in the literature. This study examined the prevalence and nature of PPI in paediatric pragmatic trials and lessons learned from researchers’ experiences. Methods: For this mixed methods study, we conducted an online survey and semi-structured interviews with corresponding authors of published paediatric pragmatic trials, identified using an online search filter in MEDLINE. Descriptive statistics and qualitative thematic analysis were used to analyse the data. Results: PPI was reported by 71/127 (56%) survey respondents. Reported impacts of PPI in the survey included the following: more feasible interventions (71%), higher-quality research (57%), improved recruitment and retention (57%), and increased applicability of research findings (57%). Both survey and interview participants identified that insufficient resources, time, and training for relationship development were challenges to PPI in paediatric trials. Three themes were identified from the semi-structured interview data (recruitment and engagement, sustaining PPI relationships, and PPI value added). Conclusions: PPI aligns with the purpose and intended impact of pragmatic trials, and paediatric researchers perceive that PPI leads to increased research relevance, quality, and dissemination. There is, however, a need for institutional and funding bodies to invest in PPI partnership, including offering support for researchers and providing opportunities for children, youth, and parents as PPI partners.

## 1. Introduction

Pragmatic randomized controlled trials aim to evaluate interventions under real-world conditions and have been used to inform broad-scale clinical or public health strategies in large and diverse populations [1,2,3,4]. Pragmatic trials often involve research questions and adaptive methods suited to applied settings, and measure outcomes relevant to implementation (e.g., acceptability, feasibility, efficiency) to inform timely, equitable healthcare delivery [5]. This focus on applicability often influences design decisions, such as ensuring that interventions are feasible, eligibility criteria are broad, research materials are accessible, and outcomes are relevant and meaningful to patients and healthcare providers. Additional considerations apply to trials involving children, where barriers to participation (e.g., parental uncertainty about providing proxy consent, challenges with family routines, and educational commitments) can influence recruitment and retention, participant diversity, and generalizability of results [6,7,8].

Optimizing the “fit” of a research study with the people who participate in it and to whom the results matter most aligns with the aims of patient and public involvement (PPI) in health research, where patients, caregivers, and members of the public work alongside researchers to co-design, conduct, and share study findings [2,9]. Given the emphasis on applicability to real-world settings, PPI is often a feature of pragmatic trials [10,11], and available guidance emphasizes incorporating patient and public perspectives from the earliest stages [12,13]; for example, the PRagmatic Explanatory Continuum Indicator Summary (PRECIS-2) tool recommends incorporating patient-important outcomes [14], and the 2025 version of the CONsolidated Standards Of Reporting Trials (CONSORT) reporting guideline includes a specific item on PPI, though it is not specific to pragmatic trials [15].

In recent years, PPI has become increasingly common in child health research [16]; however, PPI is still infrequently conducted or reported in paediatric randomized trials [17]. Despite the congruency between pragmatic trials and PPI [18,19], involvement of patients and the public in paediatric pragmatic trials (and discussion of challenges and strategies for success) remain poorly reported in the literature. As a result, there are limited data on strategies to enable PPI; build trusting relationships with children, youth, parents, and other partners; and reduce barriers in the face of competing priorities [20,21]. Exploring when PPI takes place, who is involved and how, and gathering firsthand perspectives about the impact of PPI can inform the development of guidance to support authentic and meaningful PPI in paediatric pragmatic trials.

The main objectives of this research were to (1) describe PPI practice in published paediatric pragmatic trials including why, how, and when PPI was conducted, who was involved, perceived benefits, challenges, and if PPI partners were acknowledged; and (2) explore in-depth experiences of researchers, including perceived impacts and lessons learned when conducting PPI in paediatric pragmatic trials.

## 2. Materials and Methods

### 2.1. Study Design

This explanatory mixed methods study is embedded within a larger, previously published survey of corresponding authors of trials deemed likely to be pragmatic [2]. The sampling frame for this survey was randomized controlled trials published in MEDLINE [22,23], identified using a validated electronic search filter to identify trials likely to be pragmatic. Since there is no single design feature that can be used to identify trials as either pragmatic or explanatory, and reporting guidelines do not require authors to label their trials as pragmatic or explanatory, it is impossible to definitively classify trials from a large systematic review of the literature as either pragmatic or explanatory. Therefore, this filter used terms related to common designs, settings, and data sources of pragmatic trials, such as those captured in the PRECIS-2 tool [14], to identify trials that were more likely to be pragmatic.

The methods for the survey have been previously reported [2]. In brief, the online survey was sent to corresponding authors from Canada, The United States, The United Kingdom, Australia, New Zealand, South Africa, France, Belgium, Denmark, Finland, Germany, Italy, The Netherlands, Spain, Norway, Sweden, and Switzerland, as it was offered only in English. We gathered data on trial characteristics (e.g., study population, location), corresponding author characteristics (e.g., age, gender, stage of research career), whether PPI was incorporated in the trial, and, where applicable, types of PPI (e.g., who was involved, how they were involved, what were the outcomes of involvement), whether PPI partners were acknowledged or compensated, and perceived benefits and challenges of PPI.

In this study, we report survey results for the subset of trials from within the larger, previously published survey that were identified by respondents as paediatric; defined as trials with participants who were exclusively 18 years and younger, or where the primary outcome was paediatric-focused. We also report the findings from semi-structured interviews conducted with a purposive sample of the paediatric trial survey respondents who were willing to be contacted for interview. Our study is reported according to the Good Reporting of A Mixed Methods Study (GRAMMS) Checklist for mixed methods studies (see Supplementary Material S3) [24].

We included 2 PPI partners (AH, MS) in our team who had lived experience of partnership in pragmatic trial research to ensure our study objectives, data collection, and interpretation of findings reflected and aligned with the perspectives of people with health research engagement experience. These PPI partners helped conceptualize the study, contributed to the protocol and data collection tools, interpreted study findings, and co-authored the manuscript. We used the Guidance for Reporting Involvement of Patients and the Public (GRIPP2) reporting checklist [25] (see Supplementary Material S4).

### 2.2. Data Collection

**Figure 1:**
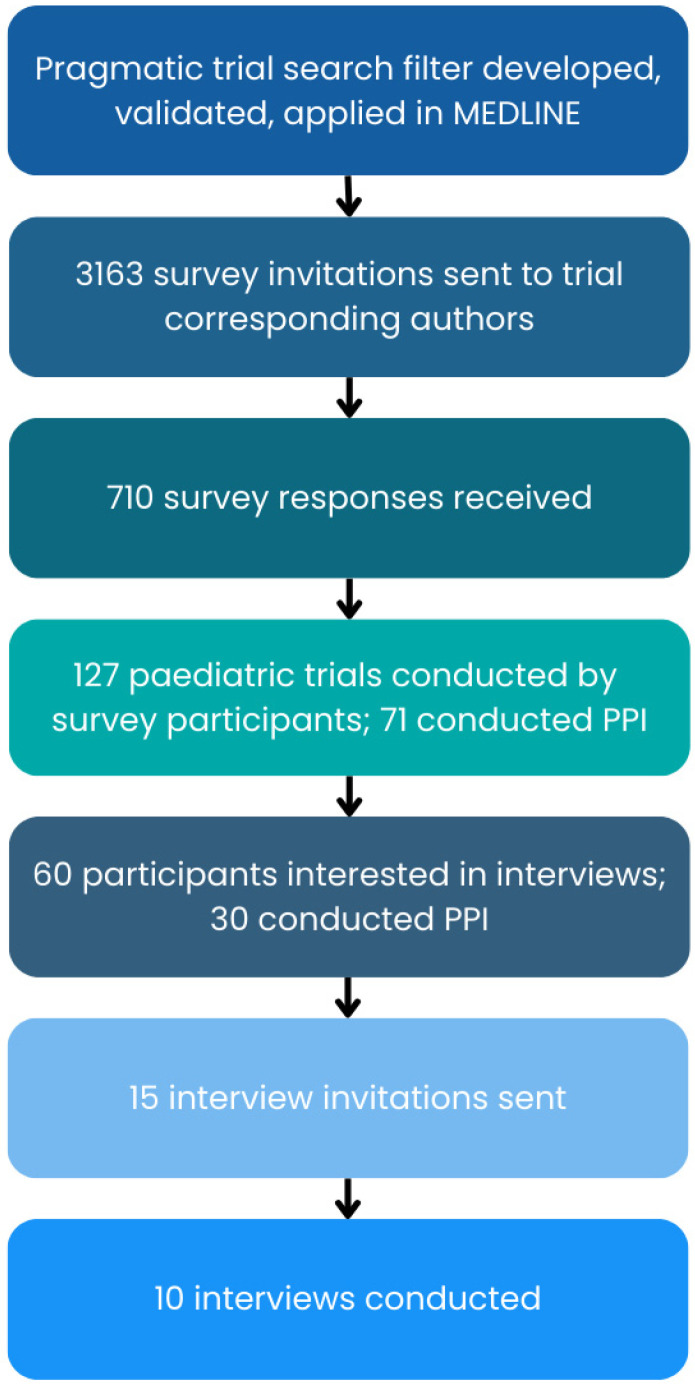
Study schematic [2,22,23].

A study schematic is shown in Figure 1. The survey questionnaire (see Supplementary Material S1) was administered based on a modified version of Dillman’s Tailored Design Method, including personalized invitations, a visually appealing survey, incentives, and three reminders. The content was based on previous published surveys on PPI [26,27], the research team’s PPI experience, and the perspectives of our PPI partners. The survey was conducted using SurveyMonkey (https://www.surveymonkey.com/, accessed on 1 February 2022), and potential participants received a personalized email containing the trial citation (authors, title, journal, year) and a unique survey link for their trial. At the start of the survey, participants reviewed an information sheet, were notified that the survey was voluntary and that continuing the survey implied consent, asked to review a definition of PPI and indicate that they had read and understood it. Survey participants were entered into a draw for a CAD 100 Amazon gift card.

Corresponding authors of paediatric trials were also given the opportunity to participate in an in-depth interview. To gather a range of perspectives, we purposively recruited participants who represented trials with children of different ages, clinical populations, and settings and collected data until saturation was achieved and no new concepts or themes were expressed [28]. All participants reviewed an information sheet prior to the interview and provided verbal consent. Interviews were conducted virtually by SV, a female qualitative researcher, and followed a semi-structured topic interview guide (see Supplementary Material S2). Interview guides were developed based on the available literature [26,27,29,30], plus the research teams’ expertise, and included questions about specific considerations and challenges with PPI in paediatric pragmatic trials, identifying and building relationships with PPI partners, and perceived impacts of PPI on trial methods, outcomes, and patients and families. Interviews were audio-recorded and transcribed verbatim by Microsoft Teams, checked for accuracy by the interviewer, and imported into NVivo v11 for analysis. An honorarium of CAD 100 was offered to interview participants.

This study was approved by the Ottawa Health Science Network Research Ethics Board (20210684-01H). Informed consent was obtained from all subjects involved in the study.

### 2.3. Analysis

Survey data were exported from SurveyMonkey into a spreadsheet via Airtable. Quantitative survey data on trial characteristics were summarized using descriptive statistics. Open-ended survey responses were collated and independently reviewed (by SV, PN, SN, MT) and classified into categories. Disagreements were resolved through discussion. Examination of the semi-structured interview transcripts followed a thematic analysis approach, in which textual data were coded and labeled in an inductive manner [31,32]. Interviews were dual-coded by SV and ST; both researchers independently reviewed each transcript and generated and discussed initial codes based on experiences and perspectives. Through discussion and consensus, the codes were then used to develop themes that best represented the perspectives shared, based on an iterative process using the constant comparison method [33].

## 3. Results

### 3.1. Survey

Of 3163 online survey invitations sent, we received 710 (28%) responses, and 127 (18%) of these were from corresponding author respondents of paediatric trials (see Figure 1). Of these 127 paediatric trials, 71 (56%) had PPI in the trial. Of the 30 corresponding authors of paediatric trials that expressed an interest in participating in a semi-structured interview, 10 interviews were conducted.

Table 1 describes survey respondent characteristics for the paediatric trials. Respondents predominantly identified as women (63%), from the USA (48%), in their mid (23%) or late (67%) academic career, and over half reported more than a decade of experience with PPI in research (59%).

PPI was reported in 56% (71) of paediatric trials. As shown in Table 2, PPI partners were commonly identified through previous collaborations (46; 74%) and were often parents or caregivers (28; 42%) or children under 18 years of age (25; 37%). PPI partners most commonly supported the design and development of interventions (45; 68%), contributed to the development of recruitment and retention strategies (41; 62%), and helped troubleshoot issues (27; 41%). A range of PPI partner preparation methods were provided, as follows: 65% (43) received written materials, 61% (40) were provided orientation sessions, and 58% (38) were offered discussions about mutual expectations. PPI partners were compensated for their time in approximately half (32; 49%) of trials, and the prevalence of PPI partner acknowledgement in trial publications was 58% (37).

As shown in Table 3, respondents reported that the predominant reasons for incorporating PPI were increased research relevance (55; 82%), quality (48; 72%), and dissemination (35; 52%), along with a moral imperative (38; 57%). Almost 90% (55) reported benefits of PPI, including more feasible interventions (44; 71%), higher-quality research (35; 57%), improved recruitment and retention (35; 57%), and increased applicability of the findings (35; 57%). Fewer than half of respondents reported challenges, the most common of which were recruiting PPI partners (13; 20%) and scheduling meetings (13; 20%).

### 3.2. Semi-Structured Interviews with Paediatric Trialists

Of the 30 corresponding authors of paediatric trials with PPI that expressed an interest in participating in semi-structured interviews, we conducted interviews with 11 researchers representing 10 trials (one interview had two participants; see Figure 1 and Supplementary Material S5 for participant characteristics). Interviews lasted from 25 to 61 min. Participants were residents in the United States (n = 6), Canada (n = 1), and the United Kingdom (n = 4). Trial interventions included school- and community-based programs to promote healthy eating, active living, and sexual health, support groups for parents of children with mental health needs, and mobile health interventions for children with chronic pain. We identified three ‘lessons learned’ themes from the interview data:

(a) Recruitment and engagement of PPI partners

Interview participants recounted challenges with recruiting an inclusive group of PPI partners. They postulated that some people may not have been able to partner on their trials due to insufficient time or other capacity constraints; this led to PPI partners often having specific characteristics, such as being experienced in health research or retired. This contrasted with most trial participant target populations—children and youth, busy caregivers of young children, and people of lower socioeconomic status—who had limited time and required support (e.g., age-appropriate resources to introduce them to health research or caregiving) to fully participate as PPI partners. With such target populations, sharing opportunities about PPI, securing interest and commitment, and finding times and spaces that were accessible and convenient posed challenges for researchers. Though five of the ten trials involved children and youth as PPI partners, engagement in these settings was described as consultation or large group discussions about feasibility or relevance, whereas, for paediatric trials involving adult (either caregivers of children or adult members of the public) PPI partners, these PPI partners more often took on co-design or decision-making roles within the research team.

*So, for us the path of least resistance is [involving people who are well positioned for PPI but may not represent the trial target population]. They’re the ones who are willing to come forward and saying, ‘Yep, I’m available, you know, twice a month and I’ll speak with you, no problem.’ So the people on the ground, which I would say. The ones we need to talk to are harder to find, and that’s because we don’t look in the right places. We don’t have time as academics sometimes to work up relationships with organizations who are already interacting and serving with our target PPI people. Interview participant 2*

(b) Sustaining PPI Relationships

The recruitment challenge also raised the issue that some researchers felt poorly equipped or lacked sufficient resources and skills to establish and maintain trusting relationships with PPI partners (e.g., children and youth), especially when the concept of PPI was new to all parties.

*But then that requires a whole different skill set, time, and effort. Like it’s one thing to say, OK, we need patients and the public to help us design our research methods. So that’s something that’s new to me personally and many of my peers. We have not been trained for it in our PhDs, you know … we are just … scrambling around … just trying to act on what we think is the right thing to do without much evidence sometimes. Interview participant 2*

(c) PPI value: more than the sum of the parts

Several interview participants perceived that PPI added value to the research, to their own skills and perspectives, and to the PPI partners; these benefits were often described as exceeding their expectations. Researchers described PPI relationships as key to enhancing the impact and rigor of their trials, but also important for stimulating research ideas, enhancing the skills of the research team, their sense of purpose as researchers, and providing motivation to conduct PPI in future research. Numerous interview participants reported that PPI partners told them that they felt heard, validated, and that their perspectives mattered. One trialist described a PPI partner who felt so positively about their experience that they made a video about the importance of PPI contributions to research, which was then used to help recruit other PPI partners. These impacts seemed most likely when researchers saw the value of a reciprocal approach where they provided incentives, skill-building, or educational opportunities for their PPI partners:

*One thing I haven’t mentioned, which I think is probably worth mentioning, is that the work that the young mums did with us, they also use that themselves to support their own educational development. It was like a mini project that we doing with them, you know developing a poster that could be used as our main recruitment poster in clinics, in maternity clinics and because this was an education class, initially they were using these activities and the product of their activities as part of their educational record as well. So that’s not so much about a benefit for the trial, but it is actually about demonstrating that the activity was beneficial for them as research partners, members of the public taking part in this research. Interview participant 8*

## 4. Discussion

### 4.1. Summary of Findings

Of 127 paediatric pragmatic trialists, just over half reported incorporating PPI. Paediatric researchers perceived that PPI improved intervention feasibility, research quality, recruitment and retention, applicability of findings, and relationships with PPI partners. Both the survey and interview data indicated that researchers did not always feel comfortable or able to fully engage children, youth, and caregivers as partners.

### 4.2. Comparison with Other Studies

Our findings that PPI in paediatric trials had multiple impacts on researchers, PPI partners, and the conduct and outcome of the research itself align with the other literature [34,35]. For example, other authors have shown that PPI partners often report a stronger understanding of research, healthcare systems, their own health or their child’s health, and a greater sense of community, belonging, or solidarity with people who have had similar healthcare experiences [16,21,34,35,36]. Researchers and clinicians who integrate PPI into their studies have also reported better relationships with patients and families, as well as an improved ability to deliver patient-centered healthcare, but acknowledge that significant time, resources, and expertise are needed to conduct PPI effectively [21,34,35]. Specific to pragmatic trials, other studies have identified that PPI partners provide valuable insights for communicating the relevance and importance of the trial to potential participants, enabling the recruitment of diverse participants, and support post-study implementation efforts, such as policy change [10,13,37]. The challenges reported in our sub study also corroborate similar published findings, especially around short grant application timelines, extra time needed to conduct PPI, and skill development to effectively establish trusting relationships with PPI partners, including children, youth, and parents [13,37,38]. Although our study was not designed for formal comparisons to the total sample, our findings regarding why, when, and how often PPI was conducted, how PPI contributed to the research process, and researchers’ perceptions about challenges, benefits, and impacts of PPI in paediatric trials align with our previously reported results [2]. The reported prevalence of PPI in our survey sample was higher than what has been documented in the other literature [17,39,40], which may be related to the relatively high proportion of our survey respondents who had at least 10 years of PPI experience, and this demographic may have also been more inclined to participate in our study. We previously identified that PPI is often not reported in pragmatic trial publications [2], but updated guidance from CONSORT [15] may improve this discrepancy in the future and allow for a better understanding of the true prevalence of PPI.

Some interview participants reflected on the nature of PPI in paediatric research, where children and youth are not always involved, but rather, represented by adults. The idea of involving children and youth was sometimes daunting to researchers because of ethical considerations or creating age-appropriate environments for dialogue and interactivity [16]. Often, parents, caregivers, and teachers take on advocacy roles to support the health and wellbeing of children, which lends well to the concept of PPI in research. Researchers may feel more equipped and comfortable with involving adults as PPI partners, which may be appropriate for studies about infants or parenting interventions; however, it is less so for trials involving youth. Building longstanding relationships with children and youth can also be a challenge, where their interest or capacity to engage may change over time as they progress through different stages of childhood and adolescence [38]. We also identified that, when children and youth were involved, they less frequently took on decision-making roles with equal power to other team members, such as participating in discussions around ethics and data governance; these issues have previously been reported [16,34,35,41]. To address this and diversify PPI partners [42], there is a need to build capacity for children and youth to learn about PPI and how to contribute to health research across different levels of involvement [43,44]. Involving children and youth in research may require creativity and resources such as experienced facilitators, along with flexibility to meaningfully involve and introduce complex topics [45]. There are several examples in the literature of how researchers have made PPI accessible for children and youth by establishing trusting relationships, providing skill-building opportunities towards empowerment, and offering multiple modes of participation [35,45,46,47,48,49]. However, guidance to support researchers as they navigate identifying, preparing, and providing a range of opportunities (appropriate to age, research context, and engagement capacity) for children, youth, and parents is needed. Guidelines could also encourage researchers to evaluate and document approaches, outcomes, and impacts of PPI in paediatric trials to inform best practices and ensure that, when PPI is conducted, its intended aims are likely to be achieved.

### 4.3. Strengths and Limitations

Our study has several strengths. To our knowledge, this is the first study to specifically explore PPI within the context of paediatric pragmatic trials. Our mixed methods approach allowed us to descriptively summarize key characteristics of PPI in paediatric trials, while gathering insights about the practical application of PPI. Semi-structured interviews with trial corresponding authors (often lead or senior researchers) provided valuable perspectives, as participants were often responsible for the funding, design, and implementation of the trials, as well as the decision to conduct PPI, and privy to challenges and tensions when competing perspectives and priorities arose. Including PPI partners in our research team added strength to the comprehensiveness and relevance of our data collection tools and ensured that our results could be used to create actionable, patient-centered strategies for improving PPI practice in paediatric pragmatic trial research.

This study also has some limitations. Though survey participants were instructed to read and confirm their understanding of a definition of PPI, it is possible that some described activities were inconsistent with our study focus. This study was conducted after the original trials were completed, which raises the possibility of recall bias, for example, if researcher perspectives on PPI had shifted based on evolving experiences and increasing familiarity with the PPI literature. Our response rate was relatively low, and response bias may have occurred, as researchers who were interested in participating in a study about PPI may have held strong opinions about PPI, either positive or negative, or had conducted PPI. In addition, survey respondents were provided with a modest financial incentive to participate, which may have skewed respondent characteristics. While we attempted to recruit a variety of participants from diverse trials, the inclusiveness of the final sample may be limited (the study was only conducted in English), potentially impacting the transferability of our findings. Finally, though researchers’ perspectives about PPI are important, we did not survey or interview PPI partners, whose insights would have contributed complementary perspectives about their experiences and helped deepen our findings.

## 5. Conclusions

As PPI is increasingly common in funding requirements, institutional policies, and researchers’ practice, this study highlights several opportunities where PPI in paediatric pragmatic trials can be improved. As much as possible, children, youth, and caregivers with lived experience relevant to the trial should be prioritized as PPI partners, and offered age-appropriate opportunities to share decision-making, research co-design, and knowledge sharing responsibilities with the broader research team. Researchers may require additional training, resources, or support to incorporate PPI into their pragmatic trials, but the findings of this study suggest that the benefits of PPI partnership often exceed researcher expectations and not only improve research design, but also empower PPI partners and validate their experiences, and strengthen researchers’ clinical understanding and motivations to conduct PPI in future work. Funding bodies and research institutions should prioritize the development of evidence-based guidelines, capacity and culture building, and designated funding opportunities to support paediatric pragmatic trial researchers to incorporate PPI.

## Figures and Tables

**Table 1:** Survey data on corresponding author characteristics (N = 127).

Corresponding Author Characteristic	Frequency (%)
**Region of residence**	**(n = 120)**
USA	57 (47.5)
UK	24 (20.0)
Non-UK Europe	16 (13.3)
Australia or New Zealand	9 (7.5)
Canada	9 (7.5)
Other	5 (4.2)
**Age (years)**	**(n = 118)**
<35	1 (0.8)
36–45	23 (19.5)
46–55	41 (34.7)
56–65	38 (32.2)
>65	15 (12.7)
Prefer not to answer	1 (0.8)
**Gender**	**(n = 117)**
Man	42 (35.9)
Woman	74 (63.2)
Prefer not to disclose	1 (0.8)
**Stage of research career**	**(n = 118)**
Early career (within 5 years of first academic appointment)	9 (7.6)
Mid-career (6–15 years since first academic appointment)	27 (22.9)
Late career (>15 years since first academic appointment)	79 (66.9)
Non-academic	3 (2.5)
**Years of PPI experience**	**(n = 118)**
<1 year	6 (5.1)
1–3 years	9 (7.6)
4–10 years	34 (28.8)
>10 years	69 (58.5)

**Table 2:** PPI characteristics of paediatric trials with PPI (N = 71).

Characteristic	Frequency (%)
How patient/public partners were identified *	**(n = 62)**
Previous collaborations	46 (74.2)
Health system partnering with other organization	22 (35.5)
Word of mouth/recommendation from colleague(s)	24 (38.7)
Community outreach/social media	19 (30.6)
From a directory	3 (4.8)
Other (e.g., matching service)	16 (25.8)
Patient/public partners *	**(n = 67)**
Children or youth (≤18 years of age)	25 (37.3)
Parents or caregivers	28 (41.8)
Adult patients (>18 years of age)	14 (20.9)
Patient advocacy group members	13 (19.4)
Members of the public	16 (23.9)
**Attempt by researchers to ensure PPI partners reflected the diversity of the trial target population**	**(n = 60)**
Yes *	41 (68.3)
Age	25 (41.7)
Race/ethnicity/culture/language	28 (46.8)
Gender	18 (30.0)
Birth-assigned sex	19 (31.7)
Place of residence	16 (26.7)
Socioeconomic status, education, religion, social capital	35 (58.3)
Other	5 (8.3)
No	19 (31.7)
PPI partner involvement in study *	**(n = 66)**
Designing or developing interventions	45 (68.2)
Developing recruitment or retention strategies	41 (62.1)
Designing recruitment materials	28 (42.4)
Suggesting dissemination strategies	22 (33.3)
Participating in the Trial Steering Committee	19 (28.8)
Selecting outcomes	11 (16.7)
Developing data collection tools	23 (34.8)
Setting research topics or questions	19 (28.8)
Presenting findings to a lay audience	20 (30.3)
Troubleshooting issues	27 (40.9)
Writing or reviewing lay summaries	17 (25.8)
Writing or reviewing manuscripts	13 (20.0)
Identifying or screening potential participants	14 (21.2)
Collecting data	17 (25.8)
Determining target difference or statistical analysis plan	5 (7.6)
Analyzing or interpreting qualitative or quantitative data	21 (31.8)
Participating in the Data Safety Monitoring Board	4 (6.1)
Preparing presentations for scientific conferences	11 (16.7)
Other (e.g., missing data handling, delivering intervention)	4 (6.1)
Preparation of patient/family partners *	**(n = 66)**
Written materials about the study	43 (65.2)
Orientation sessions	40 (60.6)
Discussion of mutual expectations for involvement	38 (57.6)
Terms of reference	10 (15.2)
Research or PPI training	15 (22.7)
None	1 (1.5)
Input from PPI partners *	**(n = 66)**
Face-to-face or virtual meetings	63 (95.5)
Email or online forums	28 (42.4)
Surveys	11 (16.7)
Telephone calls/interviews	3 (4.5)
**PPI partner compensation**	**(n = 65)**
Yes	32 (49.2)
No	27 (41.5)
Don’t know or other	6 (9.2)
PPI partner acknowledgement *	**(n = 64)**
Named in acknowledgements	37 (57.8)
PPI partners did not wish to be acknowledged	4 (6.3)
Named as individual co-authors	14 (21.9)
Included in group authorship	2 (3.1)
No	27 (42.2)

* More than one selection possible.

**Table 3:** Rationale, benefits, and challenges related to PPI in paediatric trials (N = 71).

Characteristic	Frequency (%)
Reason for involving PPI partners in study *	**(n = 67)**
Increased applicability/relevance of research	55 (82.1)
Increased quality of research	48 (71.6)
Increased dissemination/uptake of findings	35 (52.2)
Morally or ethically the right thing to do	38 (56.7)
Funding body requirement/recommendation	17 (25.4)
Increased feasibility or quality of intervention	3 (4.5)
Institutional requirement/recommendation	12 (17.9)
Target journal requirement/recommendation	2 (3.0)
Other	4 (6.0)
**Benefits of involving patient/public partners**	**(n = 62)**
Yes *	55 (88.7)
Improved/more feasible interventions	44 (71.0)
Increased applicability/relevance of findings	35 (56.5)
Higher-quality research	35 (56.5)
Improved recruitment or retention	35 (56.5)
Enhanced relationships/networking with partners	21 (33.9)
Enhanced understanding of condition	18 (29.0)
Increased dissemination or uptake of results	18 (29.0)
Increased participant satisfaction	22 (35.5)
Increased satisfaction of research team	19 (30.6)
Increased accountability or public trust in research	21 (33.9)
Led to identifying knowledge gaps or future research topics	17 (27.4)
More ethically acceptable methods	24 (38.7)
More useful evidence for patients	13 (21.0)
More useful evidence for decision-makers	15 (24.2)
Led to collaboration on other studies	17 (27.4)
Improved data quality	14 (22.6)
Increased funding opportunities	8 (12.9)
No	7 (11.3)
**Challenges experienced**	**(n = 65)**
Yes *	31 (47.7)
Communicating about trial design, methods, results	12 (18.5)
Identifying or recruiting PPI partners	13 (20.0)
Scheduling meetings	13 (20.0)
Sustaining involvement of PPI partners throughout the study	12 (18.5)
Clarifying roles and expectations	12 (18.5)
Time commitment	11 (16.9)
Building relationships with PPI partners	6 (9.2)
Costs	5 (7.7)
Managing conflicts	6 (9.2)
Study timeline extended	5 (7.7)
Compensation	2 (3.1)
Representation of population of interest	1 (1.5)
Other	7 (10.8)
No	34 (52.3)

* More than one selection possible.

## Data Availability

Data are available upon request to the corresponding author due to institutional privacy and ethics policies.

## Supplementary Materials

The following supporting information can be downloaded at: https://www.mdpi.com/article/10.3390/children12121638/s1, Supplementary Material S1: Survey Questionnaire; Supplementary Material S2: Interview Guide; Supplementary Material S3: Good Reporting of A Mixed Methods Study (GRAMMS) Checklist for mixed methods studies; Supplementary Material S4: Guidance for Reporting Involvement of Patients and the Public (GRIPP2) reporting checklist; Supplementary Material S5: Interview Participant Characteristics.
