# Supplementary material for: Patient and Public Involvement in Paediatric Pragmatic Randomized Controlled Trials: A Mixed Methods Study
Source: Children (Basel). 2025 Dec 2;12(12):1638. doi: 10.3390/children12121638 (PMC12731746; doi:10.3390/children12121638)
Supplement: Supplementary file 1 [file children-12-01638-s001.zip › Supplementary Material S1.pdf]

Patient/Public Involvement in Your Randomized Controlled Trial

Implied Consent Form

OHSN-REB Number: 20210684-01H
Principal Investigator: Dr. Monica Taljaard; 613-798-5555 X18618
Sponsor: Ottawa Hospital Research Institute

INTRODUCTION

You are invited to participate in an online survey for a research study. You are receiving this invitation because you are the corresponding author for a published randomized controlled trial selected from MEDLINE. This consent form provides you with information to help you make an informed choice. Please read this document carefully and ask any questions you may have. All your questions should be answered to your satisfaction before you decide whether to participate. Please take your time in making your decision. Taking part in this study is voluntary. Deciding not to take part or deciding to leave the study later will not result in any penalty.

IS THERE A CONFLICT OF INTEREST?

There are no conflicts of interest to declare related to this study.

WHAT WILL HAPPEN DURING THIS STUDY?

You will proceed to an online questionnaire about patient and public involvement in your study, which will take about 5-10 minutes to complete. The information you provide is for research purposes only. Some of the questions are personal. You can choose not to answer questions if you wish.

VOLUNTARY PARTICIPATION AND WITHDRAWAL

You do not have to be in this study if you do not want to be. You can choose to end your participation at any time without having to provide a reason. If you decide to withdraw, you can ask that the information that was collected about you not be used for the study. Let the research team know if you choose this.

RISKS AND/OR BENEFITS

You can choose not to answer questions that make you feel uncomfortable. You may not receive any direct benefit from your participation in this study. We hope that findings from this study will help researchers conduct patient and public involvement in their randomized controlled trials more effectively in the future. If you decide to participate in this study, the research team will only collect the information they need for this study.

S2, as supplied by the authors. Appendix to: Vanderhout S, Nevins P, Nicholls SG, et al. Patient and public involvement in pragmatic trials: online survey of corresponding authors of published trials. CMAJ Open 2023. doi: 10.9778/cmajo.20220198.
Copyright © 2023 The Author(s) or their employer(s). To receive this resource in an accessible format, please contact us at cmajgroup@cmaj.ca.

PRIVACY/CONFIDENTIALITY

Records identifying you at this centre will be kept confidential and, to the extent permitted by the applicable laws, will not be disclosed or made publicly available, except as described in this consent document. Authorized representatives of the following organizations may look at your original (identifiable) records at the site where these records are held, to check that the information collected for the study is correct and follows proper laws and guidelines: *The Ottawa Health Science Network Research Ethics Board* who oversees the ethical conduct of this study; *Ottawa Hospital Research Institute*, to oversee the conduct of research at this location.

Information collected about you for the study may also be sent to the organizations listed above. Your name, address, or other information that may directly identify you will not be used. The records received by these organizations may contain your participant code.

Communication via e-mail is not absolutely secure. We do not recommend that you communicate sensitive personal information via e-mail. If the results of this study are published, your identity will remain confidential. It is expected that the information collected during this study will be used in analyses and will be published / presented to the scientific community at meetings and in journals. Your de-identified data from this study may be used for other research purposes. If your study data are shared with other researchers, information that links your study data directly to you will not be shared. If information from this study is published, shared, or presented at scientific meetings, your name and other personal information will not be used. Even though the likelihood that someone may identify you from the study data is very small, it can never be completely eliminated.

COST AND/OR PAYMENT

You will not be paid for taking part in this study. If you choose to participate, to thank you for your time, you will be entered into a draw for one of five $100 CAD Amazon gift cards.

RIGHTS OF PARTICIPANTS

You will be told, in a timely manner, about new information that may be relevant to your willingness to stay in this study.

You have the right to be informed of the results of this study once the entire study is complete. If you would like to be informed of the results of this study, please contact the research team. Your rights to privacy are legally protected by federal and provincial laws that require safeguards to ensure that your privacy is respected. By participating, you do not give up any of your legal rights against the researcher, or involved institutions for compensation, nor does this form relieve the researcher, the Ottawa Hospital Research Institute or their agents of their legal and professional responsibilities.

QUESTIONS

If you have questions about taking part in this study, please contact Dr. Monica Taljaard at mtaljaard@ohri.ca.

If you have questions about your rights as a participant or about ethical issues related to this study, you can talk to someone who is not involved in the study at all. Please contact The Ottawa Health Science Network Research Ethics Board, Chairperson at 613-798-5555 extension 16719.

CONSENT

By completing this survey, your consent to participate is implied.

S1, as supplied by the authors. Appendix to: Vanderhout S, Nevins P, Nicholls SG, et al. Patient and public involvement in pragmatic trials: online survey of corresponding authors of published trials. CMAJ Open 2023. doi: 10.9778/cmajo.20220198.
Copyright © 2023 The Author(s) or their employer(s). To receive this resource in an accessible format, please contact us at cmajgroup@cmaj.ca.

Patient/Public Involvement in Your Randomized Controlled Trial

NOTE: All questions in this survey pertain to patient or public involvement as research partners in your published randomized controlled trial identified in the email invitation.
By patient/public involvement we mean researchers consulting with or working alongside patients or members of the public and/or caregivers in all or any part(s) of the research process. This is also known as patient/public engagement, or partnering with patients/members of the public. We do NOT mean people recruited to be participants in the trial, pilot participants, or anyone solely providing some form of data for the study. We also do NOT mean policymakers or health system stakeholders.
By caregiver, we mean an "informal" caregiver such as a family member. We do NOT mean a health professional or health care provider.

* 1. Please read the definition of patient/public involvement above and check the box below. (ANSWER REQUIRED)

☐ I have read and understand the definition of patient/public involvement used in this survey

2. For your randomized controlled trial identified in the email invitation, please indicate if the trial was a paediatric trial, i.e., the trial population was restricted to children and youth (<=18 years of age) OR primary outcome was focused on paediatric (infant, child, youth) outcomes even if the randomization/intervention occurred at the level of the family/caregivers/providers/health system

◯ Yes - this was a paediatric trial

◯ No - this was not a paediatric trial

3. For your randomized controlled trial identified in the email invitation, please indicate if the trial specifically focused on older adults, i.e., recruited participants aged 65 years and older; OR recruited "older adults" using your own preferred definition (e.g., geriatric, senior, elderly) and mean or median age of participants was at least 65 years of age

◯ Yes - this was a trial of older adults

◯ No - this was not a trial of older adults

* 4. Were any patients, caregivers, or members of the public involved as partners in any aspect of the design, conduct, analysis, or dissemination of your study? (ANSWER REQUIRED)

◯ Yes

◯ No

◯ Don't know (please explain)

S1, as supplied by the authors. Appendix to: Vanderhout S, Nevins P, Nicholls SG, et al. Patient and public involvement in pragmatic trials: online survey of corresponding authors of published trials. CMAJ Open 2023. doi: 10.9778/cmajo.20220198.
Copyright © 2023 The Author(s) or their employer(s). To receive this resource in an accessible format, please contact us at cmajgroup@cmaj.ca.

Patient/Public Involvement in Your Randomized Controlled Trial

5. Why did you not involve patient/public partners in your study? Please select all that apply.

- ☐ Insufficient knowledge of how to involve patient/public partners
- ☐ Lack of resources or funds
- ☐ Too burdensome (time and/or costs)
- ☐ Insufficient evidence of effectiveness
- ☐ No requirement to do so
- ☐ This trial did not involve patients or members of the public as research participants
- ☐ Did not seem relevant
- ☐ Don’t know
- ☐ Other (please specify)

6. Please provide more information about why patient/public involvement was not included in your study. For example, if it did not seem relevant, please explain why you thought so.

S1, as supplied by the authors. Appendix to: Vanderhout S, Nevins P, Nicholls SG, et al. Patient and public involvement in pragmatic trials: online survey of corresponding authors of published trials. CMAJ Open 2023. doi: 10.9778/cmajo.20220198.
Copyright © 2023 The Author(s) or their employer(s). To receive this resource in an accessible format, please contact us at cmajgroup@cmaj.ca.

Patient/Public Involvement in Your Randomized Controlled Trial

7. Why did you choose to involve patient/public partners in your study? Please select all that apply.

- ☐ Increased quality of research
- ☐ Increased applicability / relevance of research
- ☐ Increased dissemination / uptake of research findings
- ☐ Institutional requirement / recommendation
- ☐ Funding body requirement / recommendation
- ☐ Target journal requirement / recommendation
- ☐ Morally or ethically the right thing to do
- ☐ Don’t know
- ☐ Other (please specify)

8. How did you identify patient/public partners to involve in your study? Please select all that apply.

- ☐ Word of mouth / recommendation from colleague(s)
- ☐ From a directory (e.g., a disease registry where people consent to being contacted to partner with researchers)
- ☐ Community outreach / social media
- ☐ Health system partnering with other organization (e.g., advocacy group)
- ☐ Previous collaborations (e.g., networks, previous studies)
- ☐ Through a matching service set up by an institution
- ☐ Don't know
- ☐ Other (please specify)

9. How many patient/public partners were involved, in total, across all stages of your study?

S1, as supplied by the authors. Appendix to: Vanderhout S, Nevins P, Nicholls SG, et al. Patient and public involvement in pragmatic trials: online survey of corresponding authors of published trials. CMAJ Open 2023. doi: 10.9778/cmajo.20220198.
Copyright © 2023 The Author(s) or their employer(s). To receive this resource in an accessible format, please contact us at cmajgroup@cmaj.ca.

10. Please indicate which of the following types of patient/public partners were involved in your study. Please select all that apply.

- [ ] Adult patients (>18 years of age)
- [ ] *Older* adult patients (>65 years of age, or other definition of "older" preferred by the authors)
- [ ] Caregivers of adult patients (e.g., caregiving spouse, family member)
- [ ] Caregivers of *older* adult patients (e.g., caregiving spouse, family member)
- [ ] Members of the public
- [ ] Parents or caregivers of children or youth
- [ ] Children or youth (<=18 years of age)
- [ ] Patient advocacy group members (i.e., members of an association that concerns itself with helping people or families affected by a medical condition)
- [ ] Don't know
- [ ] Other (please specify)

11. Did your patient/public partners have lived experience of the clinical condition or disease targeted in your trial?

- ( ) Yes, all
- ( ) Yes, at least some
- ( ) No
- ( ) Not applicable - the trial was not targeting a specific clinical condition
- ( ) Other (please explain)

12. Did you attempt to ensure that patient/public partners reflected the diversity of the trial's target population (e.g., by ethnicity, gender, education, etc.)?

- ( ) Yes
- ( ) No
- ( ) Don't know or Other (please explain)

S1, as supplied by the authors. Appendix to: Vanderhout S, Nevins P, Nicholls SG, et al. Patient and public involvement in pragmatic trials: online survey of corresponding authors of published trials. CMAJ Open 2023. doi: 10.9778/cmajo.20220198.
Copyright © 2023 The Author(s) or their employer(s). To receive this resource in an accessible format, please contact us at cmajgroup@cmaj.ca.

Patient/Public Involvement in Your Randomized Controlled Trial

13. Which of the following diversity characteristics did you consider when recruiting your patient/public partners? Please select all that apply.

- ☐ Sex
- ☐ Gender
- ☐ Age
- ☐ Race/ethnicity/culture/language
- ☐ Occupation (including employment status)
- ☐ Place of residence (e.g., urban/suburban/rural; countries; regions)
- ☐ Religion
- ☐ Education
- ☐ Socioeconomic status
- ☐ Social capital (i.e., strength of relationships, support networks, housing insecure)
- ☐ Other (please specify)

S1, as supplied by the authors. Appendix to: Vanderhout S, Nevins P, Nicholls SG, et al. Patient and public involvement in pragmatic trials: online survey of corresponding authors of published trials. CMAJ Open 2023. doi: 10.9778/cmajo.20220198.
Copyright © 2023 The Author(s) or their employer(s). To receive this resource in an accessible format, please contact us at cmajgroup@cmaj.ca.

Patient/Public Involvement in Your Randomized Controlled Trial

14. At what time/stage in the research process were patient/public partners first involved?

- ○ Pre-protocol stage (e.g., priority-setting, identifying the research question)
- ○ Protocol development stage
- ○ Trial conduct stage
- ○ Analysis stage
- ○ Interpretation of results stage
- ○ Dissemination of findings stage
- ○ Don't know
- ○ Other (please specify)

15. How did you prepare patient/public partners to be involved in your study? Please select all that apply.

- ☐ Held orientation meetings to explain the study
- ☐ Provided partners with written materials about the study
- ☐ Discussed mutual expectations for involvement
- ☐ Prepared terms of reference for engagement
- ☐ Provided partners with training in research (e.g., trial designs, research methods)
- ☐ Provided partners with training in patient/public engagement
- ☐ Don’t know
- ☐ Other (please specify)
- ☐ None of the above

S1, as supplied by the authors. Appendix to: Vanderhout S, Nevins P, Nicholls SG, et al. Patient and public involvement in pragmatic trials: online survey of corresponding authors of published trials. CMAJ Open 2023. doi: 10.9778/cmajo.20220198.
Copyright © 2023 The Author(s) or their employer(s). To receive this resource in an accessible format, please contact us at cmajgroup@cmaj.ca.

16. What methods were used to engage with patient/public partners in your study? Please select all that apply.

- [ ] Face to face meetings
- [ ] Virtual (online) meetings
- [ ] Email consultations (e.g., invited partners to comment on protocol, data collection tools, manuscripts etc.)
- [ ] Online forums (i.e., online discussion sites where people can have conversations in the form of posted messages)
- [ ] Surveys
- [ ] Don’t know
- [ ] Other (please specify)

S1, as supplied by the authors. Appendix to: Vanderhout S, Nevins P, Nicholls SG, et al. Patient and public involvement in pragmatic trials: online survey of corresponding authors of published trials. CMAJ Open 2023. doi: 10.9778/cmajo.20220198.
Copyright © 2023 The Author(s) or their employer(s). To receive this resource in an accessible format, please contact us at cmajgroup@cmaj.ca.

Patient/Public Involvement in Your Randomized Controlled Trial

17. In which specific aspects of your study were patient/public partners involved? Please select all that apply.

- ☐ Setting research topics or questions
- ☐ Designing or developing interventions
- ☐ Selecting the primary or secondary outcome(s)
- ☐ Developing recruitment strategies
- ☐ Developing retention strategies
- ☐ Designing participant recruitment materials (information sheets, consent forms, recruitment advertisements)
- ☐ Developing data collection tools (questionnaires, interview schedules)
- ☐ Determining the target difference (clinically meaningful difference or non-inferiority margin) for sample size calculation
- ☐ Developing the statistical analysis plan
- ☐ Identifying or screening potential participants
- ☐ Collecting research data
- ☐ Troubleshooting challenges during data collection
- ☐ Participating on the Trial Steering Committee
- ☐ Participating on the Data Safety Monitoring Board
- ☐ Analyzing qualitative data
- ☐ Analyzing quantitative data
- ☐ Informing missing data handling
- ☐ Interpreting data or results
- ☐ Writing or reviewing manuscripts
- ☐ Writing or reviewing lay summaries
- ☐ Suggesting routes/platforms for dissemination
- ☐ Preparing presentations for scientific conferences
- ☐ Presenting the findings to a lay audience
- ☐ Don’t know

Other (please specify)

S1, as supplied by the authors. Appendix to: Vanderhout S, Nevins P, Nicholls SG, et al. Patient and public involvement in pragmatic trials: online survey of corresponding authors of published trials. CMAJ Open 2023. doi: 10.9778/cmajo.20220198.
Copyright © 2023 The Author(s) or their employer(s). To receive this resource in an accessible format, please contact us at cmajgroup@cmaj.ca.

Patient/Public Involvement in Your Randomized Controlled Trial

18. Were patient/public partners reimbursed for out of pocket expenses related to their involvement?

- ◯ Yes, always
- ◯ Yes, sometimes
- ◯ No
- ◯ Don’t know
- ◯ Not applicable – no expenses were incurred by patient and public partners
- ◯ Other (please specify)

19. Were patient/public partners compensated (e.g. cash, vouchers, honoraria) for their involvement?

- ◯ Yes
- ◯ No
- ◯ Don't know (please explain)

20. How were patient/public partner contributions acknowledged in the manuscript? Please select all that apply.

- ☐ Named as co-author(s)
- ☐ Included in group authorship (e.g., "on behalf of...")
- ☐ Named in acknowledgements section
- ☐ Not applicable: Patient/public partners elected not to be acknowledged
- ☐ Other (please specify)
- ☐ None of the above

S1, as supplied by the authors. Appendix to: Vanderhout S, Nevins P, Nicholls SG, et al. Patient and public involvement in pragmatic trials: online survey of corresponding authors of published trials. CMAJ Open 2023. doi: 10.9778/cmajo.20220198.
Copyright © 2023 The Author(s) or their employer(s). To receive this resource in an accessible format, please contact us at cmajgroup@cmaj.ca.

21. Were there any benefits to the research study or to the investigators from involving patient/public partners in your study?

- Yes
- No
- Don't know (please explain)

S1, as supplied by the authors. Appendix to: Vanderhout S, Nevins P, Nicholls SG, et al. Patient and public involvement in pragmatic trials: online survey of corresponding authors of published trials. CMAJ Open 2023. doi: 10.9778/cmajo.20220198.
Copyright © 2023 The Author(s) or their employer(s). To receive this resource in an accessible format, please contact us at cmajgroup@cmaj.ca.

Patient/Public Involvement in Your Randomized Controlled Trial

22. What were the benefits of patient/public involvement to the research study or to the investigators? Please select all that apply.

- ☐ Higher quality research
- ☐ Enhanced understanding of illness/condition
- ☐ Improved or more feasible interventions
- ☐ More ethically acceptable research methods
- ☐ Improved recruitment, accrual or retention
- ☐ Improved data quality
- ☐ Increased satisfaction of trial participants
- ☐ Increased translation, dissemination or uptake of results
- ☐ More useful evidence for patients
- ☐ More useful evidence for clinical or health policy decision-making
- ☐ Increased applicability / relevance of research
- ☐ Increased satisfaction of research team
- ☐ Increased accountability or public trust in research
- ☐ Led to collaboration on other studies
- ☐ Led to identifying knowledge gaps and/or future research topics
- ☐ Enhanced relationships / networking with patient partners
- ☐ Increased funding opportunities
- ☐ Other (please specify)

S1, as supplied by the authors. Appendix to: Vanderhout S, Nevins P, Nicholls SG, et al. Patient and public involvement in pragmatic trials: online survey of corresponding authors of published trials. CMAJ Open 2023. doi: 10.9778/cmajo.20220198.
Copyright © 2023 The Author(s) or their employer(s). To receive this resource in an accessible format, please contact us at cmajgroup@cmaj.ca.

Patient/Public Involvement in Your Randomized Controlled Trial

23. Were there any challenges involving patients/public partners in your study?

- ◯ Yes
- ◯ No
- ◯ Don't know (please explain)

S1, as supplied by the authors. Appendix to: Vanderhout S, Nevins P, Nicholls SG, et al. Patient and public involvement in pragmatic trials: online survey of corresponding authors of published trials. CMAJ Open 2023. doi: 10.9778/cmajo.20220198.
Copyright © 2023 The Author(s) or their employer(s). To receive this resource in an accessible format, please contact us at cmajgroup@cmaj.ca.

Patient/Public Involvement in Your Randomized Controlled Trial

24. Which of the following challenges involving patient/public partners did you encounter in your study? Please select all that apply.

- [ ] Challenges in identifying or recruiting patient/public partners
- [ ] Challenges communicating about trial design, methods, and results
- [ ] Challenges clarifying roles and expectations
- [ ] Challenges managing conflicts
- [ ] Challenges with scheduling or hosting meetings
- [ ] Challenges building relationships with patient/public partners
- [ ] Challenges in sustaining involvement of patient/public partners throughout the study
- [ ] Challenges with patient/public partner compensation
- [ ] Increased time commitment
- [ ] Increased costs
- [ ] Timeline for study extended
- [ ] Other (please specify)

25. Please indicate if you agree or disagree with the following statement:

	Strongly agree	Somewhat agree	Neither agree nor disagree	Somewhat disagree	Strongly disagree
Involving patient/public partners was a positive experience for the research team	○	○	○	○	○

S1, as supplied by the authors. Appendix to: Vanderhout S, Nevins P, Nicholls SG, et al. Patient and public involvement in pragmatic trials: online survey of corresponding authors of published trials. CMAJ Open 2023. doi: 10.9778/cmajo.20220198.
Copyright © 2023 The Author(s) or their employer(s). To receive this resource in an accessible format, please contact us at cmajgroup@cmaj.ca.

Patient/Public Involvement in Your Randomized Controlled Trial

26. Do you have any other comments about involving patient/public partners in your study or in research generally?

27. We are planning to conduct follow-up interviews (~30-60 minutes) with a subset of trial authors about specific aspects of patient/public involvement in their trials including in paediatric and elderly populations. Would you be willing to be contacted to participate in an interview? An honorarium of $100 will be offered for your participation in an interview.

- Yes
- No

S1, as supplied by the authors. Appendix to: Vanderhout S, Nevins P, Nicholls SG, et al. Patient and public involvement in pragmatic trials: online survey of corresponding authors of published trials. CMAJ Open 2023. doi: 10.9778/cmajo.20220198.
Copyright © 2023 The Author(s) or their employer(s). To receive this resource in an accessible format, please contact us at cmajgroup@cmaj.ca.

Patient/Public Involvement in Your Randomized Controlled Trial

Demographic information

In this final section, we would like to collect some descriptive information about the respondents to our survey.

28. What is (are) your country (countries) of residence? Please select all that apply.

- [ ] United States of America
- [ ] Canada
- [ ] United Kingdom
- [ ] France
- [ ] Australia
- [ ] New Zealand
- [ ] South Africa
- [ ] Belgium
- [ ] Denmark
- [ ] Finland
- [ ] Germany
- [ ] Italy
- [ ] The Netherlands
- [ ] Norway
- [ ] Spain
- [ ] Sweden
- [ ] Switzerland
- [ ] Other (please specify)

29. What is your current age?

- ( ) <25 years
- ( ) 25-35 years
- ( ) 36-45 years
- ( ) 46-55 years
- ( ) 56-65 years
- ( ) >65 years
- ( ) Prefer not to answer

S1, as supplied by the authors. Appendix to: Vanderhout S, Nevins P, Nicholls SG, et al. Patient and public involvement in pragmatic trials: online survey of corresponding authors of published trials. CMAJ Open 2023. doi: 10.9778/cmajo.20220198.
Copyright © 2023 The Author(s) or their employer(s). To receive this resource in an accessible format, please contact us at cmajgroup@cmaj.ca.

30. Would you consider yourself:

- Early career researcher (within 5 years of first academic appointment)
- Mid-career researcher (6-15 years since first academic appointment)
- Late career researcher (>15 years since first academic appointment)
- Other (please specify)

31. How many years of experience do you have in engaging with patient/public partners?

- < 1 year
- 1-3 years
- 4-10 years
- >10 years

32. What racial or ethnic group do you belong to? Please select all that apply.

- White
- Indigenous (Status and Non-status First Nations, Inuit, Metis)
- South Asian (e.g., East Indian, Pakistani, Sri Lankan)
- Chinese
- Black (e.g., Caribbean, African descent)
- Filipino
- Latin American/Hispanic
- Other (please specify)
- Arab
- Southeast Asian (e.g., Vietnamese, Cambodian, Laotian, Thai)
- West Asian (e.g., Iranian, Afghan)
- Korean
- Japanese
- Prefer not to answer

S1, as supplied by the authors. Appendix to: Vanderhout S, Nevins P, Nicholls SG, et al. Patient and public involvement in pragmatic trials: online survey of corresponding authors of published trials. CMAJ Open 2023. doi: 10.9778/cmajo.20220198.
Copyright © 2023 The Author(s) or their employer(s). To receive this resource in an accessible format, please contact us at cmajgroup@cmaj.ca.

33. What is your current gender identity? Select all that apply.

- [ ] Woman
- [ ] Man
- [ ] Transgender
- [ ] Gender non-conforming/non-binary
- [ ] Two-spirit
- [ ] Prefer not to disclose
- [ ] A gender identity not listed (please specify)

34. Please indicate whether you would like to be entered into a random draw for one of five $100 CAD Amazon gift cards. If you would like to use a different email address for this purpose, please indicate it below.

- ( ) Yes
- ( ) No

Please provide an email address if you prefer we used a different address

This is the end of the survey. Thank you for completing the survey!

S1, as supplied by the authors. Appendix to: Vanderhout S, Nevins P, Nicholls SG, et al. Patient and public involvement in pragmatic trials: online survey of corresponding authors of published trials. CMAJ Open 2023. doi: 10.9778/cmajo.20220198.
Copyright © 2023 The Author(s) or their employer(s). To receive this resource in an accessible format, please contact us at cmajgroup@cmaj.ca.
