# Supplementary material for: Patient and Public Involvement in Paediatric Pragmatic Randomized Controlled Trials: A Mixed Methods Study
Source: Children (Basel). 2025 Dec 2;12(12):1638. doi: 10.3390/children12121638 (PMC12731746; doi:10.3390/children12121638)
Supplement: Supplementary file 1 [file children-12-01638-s001.zip › Supplementary Material S2-S5.pdf]

Supplementary Material S2: Interview Guide

1. Can you tell me a little about your study and PPI within that? (WHO WHEN WHY)
2. What were the specific goals of PPI in this research?
3. Did you include children/youth, parents/caregivers, or both, and what guided that choice? Looking back would you have made the same decision?
4. Were any patient or family partners engaged at a steering committee/leadership level?
5. What was your trial population? What steps were taken to ensure that your patient/public partners reflected this population?
    a. if applicable, Were there challenges with hard-to-reach populations? Challenges with engaging children and youth? Do you think you did this well? Why/why not?
6. Did any of these have to do with the REB/IRB?
7. Are you familiar with the concept of pragmatic trial? If so, do you consider your trial to be pragmatic? Do you think pragmatic trials raise unique issues regarding PPI? What might those be?
    a. if applicable, How do you think the cluster design influenced PPI in your trial?
    b. if trial was in a low- or middle-income country (LMIC), Did the LMIC setting of the trial raise any unique considerations or challenges? What were they?
    c. If applicable, Your trial was targeting healthcare providers; how do you think this impacted your approach to PPI?
8. In your opinion, where was there impact of PPI [tailor to type of research; e.g., disease-specific vs. health policy]? On the study itself (materials, design, conduct) on the research team or on the PPI contributors (relationships with each other and the research team, experiences/understanding of disease, knowledge about research, sense of community or purpose, etc.)? [refer back to survey results as needed]
9. What would you want other researchers engaging children/families in researchers to know based on your experience?

Supplementary Material S3: Good Reporting of a Mixed Methods Study (GRAMMS) Checklist
O'Cathain A, Murphy E, Nicholl J. The quality of mixed methods studies in health services research. J Health Serv Res Policy. 2008;13(2):92-98

Item	Where in manuscript
(1) Describe the justification for using a mixed methods approach to the research question	Page 5
(2) Describe the design in terms of the purpose, priority and sequence of methods	Page 5-6
(3) Describe each method in terms of sampling, data collection and analysis	Page 6-7
(4) Describe where integration has occurred, how it has occurred and who has participated in it	Page 6-7
(5) Describe any limitation of one method associated with the present of the other method	Page 21
(6) Describe any insights gained from mixing or integrating methods	Page 21

Supplementary Material S4: GRIPP-2 Short Form

Section and topic	Item	Reported on page No
1: Aim	Report the aim of PPI in the study	6
2: Methods	Provide a clear description of the methods used for PPI in the study	6
3: Study results	Outcomes—Report the results of PPI in the study, including both positive and negative outcomes	18-19
4: Discussion and conclusions	Outcomes—Comment on the extent to which PPI influenced the study overall. Describe positive and negative effects	18-19
5: Reflections/critical perspective	Comment critically on the study, reflecting on the things that went well and those that did not, so others can learn from this experience	N/A

Staniszewska, S., Brett, J., Simera, I. *et al.* GRIPP2 reporting checklists: tools to improve reporting of patient and public involvement in research. *Res Involv Engagem* **3**, 13 (2017). https://doi.org/10.1186/s40900-017-0062-2

Supplementary Material S5: Interview participant characteristics (N=10, the additional person did not fill out the survey)

Characteristic	Frequency (%)
Region of residence	
USA	5 (50)
UK	4 (40)
Canada	1 (10)
Age (years)	
<35	1 (10)
36-45	1 (10)
46-55	2 (20)
56-65	5 (50)
Prefer not to answer	1 (10)
Gender	
Man	2 (20)
Woman	6 (60)
Prefer not to disclose	2 (20)
Stage of research career	
Early career (within 5 years of first academic appointment)	0
Mid-career (6-15 years since first academic appointment)	2 (20)
Late career (>15 years since first academic appointment)	7 (70)
Non-academic	1 (10)
Years of PPI experience	
<1 year	0
1-3 years	0
4-10 years	3 (30)
>10 years	7 (70)
